# Rate and Predictors of Hesitancy toward SARS-CoV-2 Vaccine among Type 2 Diabetic Patients: Results from an Italian Survey

**DOI:** 10.3390/vaccines9050460

**Published:** 2021-05-04

**Authors:** Federica Guaraldi, Marco Montalti, Zeno Di Valerio, Edoardo Mannucci, Besmir Nreu, Matteo Monami, Davide Gori

**Affiliations:** 1 Pituitary Unit, IRCCS Istituto delle Scienze Neurologiche di Bologna, 40139 Bologna, Italy; federica.guaraldi@yahoo.it; 2 Department of Biomedical and Neuromotor Sciences (DIBINEM), University of Bologna, 40126 Bologna, Italy; marco.montalti7@studio.unibo.it (M.M.); davide.gori4@unibo.it (D.G.); 3 Diabetes Unit, Careggi Hospital and University of Florence, 50139 Florence, Italy; edoardo.mannucci@unifi.it (E.M.); dbnreu@gmail.com (B.N.); matteo.monami@unifi.it (M.M.)

**Keywords:** vaccine hesitancy, SARS-CoV-2 vaccine, type 2 diabetes, COVID-19

## Abstract

Vaccine hesitancy (VH) has been identified as one of the major health concerns of our time by the World Health Organization. It may prove especially detrimental in the light of the ongoing SARS-CoV-2 pandemic, as vaccination campaigns still represent the primary strategy against the detrimental consequences of the pandemic. Among patients suffering from type 2 diabetes mellitus (DB), who are particularly vulnerable to COVID-19, VH might represent an even more serious threat. Therefore, our study focuses on identifying potential determinants of VH among patients with type 2 diabetes. Study participants (*n* = 1176) filled in a two-section online self-administered questionnaire, answering questions regarding demographic and anamnestic data, as well as their intention to accept any vaccination against COVID-19. Some possible reasons underlying VH were investigated as well. An overall hesitancy rate of 14.2% was registered. Data showed how older age, male gender, higher education level, and having been vaccinated for seasonal influenza in 2020–2021 were associated with a significantly higher propensity to receive the COVID-19 vaccine. On the contrary, having experienced adverse effects following past vaccinations was a negative predictor. In addition to confirming an array of predictors of VH, we found a worryingly high prevalence of VH among diabetics, who have been shown to be particularly exposed to severe COVID-19 and death. These findings may be useful in planning targeted action toward acceptance improvement and enhancing the efficacy of vaccination campaigns.

## 1. Introduction

Vaccination represents one of the most effective tools to reduce the spread and clinical severity and, potentially, eradicate communicable diseases [1]. The paramount importance of safe and efficacious vaccines has recently re-emerged, given the disastrous effects of SARS-CoV-2′s spread on healthcare and economy.

The mass vaccination campaigns we are witnessing imply remarkable logistic and organizational challenges, in the effort to reach the herd immunity threshold (which is estimated at 60–75% of the population gaining immunity [2,3]) as quickly as possible. In this context, vaccine hesitancy (VH) plays a tremendously detrimental effect by slowing down, if not completely thwarting, universal vaccination.

VH is defined as the “delay in acceptance or refusal of vaccination despite availability of vaccination services” [4] and includes all the negative attitudes toward vaccination, from utter opposition to reluctance. This phenomenon has progressively and significantly increased in the last decade, with tangible effects on vaccine acceptance rates, that have prompted the World Health Organization (WHO) to list VH among world major health concerns in 2019 [5]. The damaging potential of VH becomes particularly worrying when considering vulnerable populations, such as patients with diabetes mellitus (DM), for Sars-CoV-2.

Indeed, type 2 diabetes has been proven to be—together with cardiovascular diseases and overweight—one of the major risk factors associated with severe complications and worse prognosis [6,7,8], with an estimated case-fatality rate of 7.3% vs. 2.3% in the nondiabetic population of COVID-19 confirmed cases [9].

Although the exact mechanisms responsible for the increased vulnerability of diabetic patients to SARS-CoV-2 infection remain to be explained [10], great efforts are being done to ensure the widest vaccine coverage in these patients in the shortest possible time.

Based on these premises, our study aimed at assessing the rate and potential predictors of VH toward Sars-CoV-2 vaccination in a large cohort of Italian diabetic patients.

## 2. Materials and Methods

Data were collected using a purposely designed, self-administered questionnaire, delivered online from 1 to 28 January 2021 to Italian-speaking diabetic patients that were part of one private Facebook group (Portale Diabete) and one Facebook page (Diabete.com).

The questionnaire consisted of two sections. The first was aimed at collecting demographic—i.e., age, gender, educational level, presence of subjects aged ≥65 years in the household, geographic origin (i.e., northern, central or southern Italy); and anamnestic data of interest—i.e., previous diagnosis of type 2 diabetes, previous SARS-CoV-2 infection, previous vaccination for seasonal influenza, and adverse reactions to previous vaccinations. The second explored the subjects’ intentions to be vaccinated against COVID-19. Subjects were considered “hesitant” toward vaccination if they answered “uncertain”, “extremely”, or “somewhat unlikely to get vaccinated”. Some reasons underlying VH were investigated in the group of hesitant subjects, including fear for dangerous vaccine side effects, previous SARS-CoV-2 infection (for which the subject would refuse vs. wait for vaccination), adversity toward vaccination in general, and certified medical contraindications toward vaccination.

Variables were described as absolute and relative frequencies. Wilcoxon and chi-square tests were used to assess differences between groups for dimensional and categorical variables, respectively. A multivariate analysis was performed for vaccine hesitancy, imputing all variables significantly (*p* < 0.05) associated with VH in the univariate analysis as putative moderators, using a stepwise logistic regression model. A sensitivity analysis was performed to avoid collinearity, after excluding vaccination to seasonal influenza. Data were collected using Microsoft Excel (Microsoft Corporation). All the analyses were carried out using IBM SPSS Statistics (IBM Corp. Released 2020. IBM SPSS Statistics for Windows, Version 27.0. Armonk, NY: IBM Corp).

## 3. Results

### 3.1. Main Sample Features

The sample consisted of 1176 subjects affected by type 2 diabetes (Table 1). Fifty-five had been excluded from the original sample, as they denied informed consent to study participation.

Females (*n* = 860) represented 73.1% of the sample. The majority of the subjects were aged 41 to 60 years old (*n* = 564; 48%), while few were over 71 (*n* = 82; 7%; 8 of which over 81). Half of the respondents (*n* = 583) had a university degree and 29.8% had a post-university degree (*n* = 350), while only 1.9% (*n* = 19) had finished their educational careers before receiving a high school diploma. The majority of the patients came from central (*n* = 603; 51.3%) and southern 366 (31.1%) Italy; only 30.2% of the respondents lived with people aged over 65 years old. More than half (*n* = 812; 69%) had been vaccinated for influenza in the 2020–2021 season; a minority of the patients experienced adverse events at previous vaccinations (*n* = 142; 12.1%), with very rare severe reactions (*n* = 24; 2%). Only 2.5% of the subjects had been diagnosed with SARS-CoV-2 infection (Table 1).

### 3.2. Attitude toward COVID-19 Vaccination

At the time of the survey, only 5.1% (*n* = 60) of the respondents had received a COVID-19 vaccination. Overall, 77.9% (*n* = 916) were prone to get vaccinated, 1.5% (*n* = 18) were uncertain, while 14.2% (*n* = 167) were reluctant. Among them, 126 (75.4%) were afraid of adverse events, only 20 (1.7%) declared to be against any type of vaccination, while 19 (1.6%) had certified contraindications (Table 1).

### 3.3. Predictors of Vaccine Hesitancy (VH)

Older age (confidence values: 85.8% in people aged >61 years old vs. 80.0% in those aged 31–40; *p* < 0.001), male gender (males 87.3% vs. females 81.5%), higher education level (high school and over: 87.7%; under high school: 52.6%; *p* < 0.001) and having been vaccinated for seasonal 2020–2021 flu (vaccinated 91.9%; not vaccinated 63.5%) were associated with significantly (*p* < 0.001) higher propensity to receive anti-COVID-19 vaccine (Table 2). On the other hand, having experienced adverse effects following previous vaccinations was a negative predictor (72.5% vs. 87.3%; *p* < 0.001). Finally, the geographic origin, the presence of subjects aged ≥65 years old in the household, and previous SARS-CoV-2 infection did not influence the VH rate.

According to the multivariate analysis, vaccination for seasonal 2020–2021 flu (OR = 0.16, *p* < 0.001, 95% CI = 0.11–0.22), while if compared to respondents without a high school diploma, those with a higher level of education showed a higher propensity toward vaccination. In particular, those with a high school diploma (OR = 0.21, *p* = 0.007, 95% CI = 0.06–0.66) and university education (OR = 0.15, *p* = 0.001, 95% CI = 0.05–0.47) had decreased odds of reporting hesitancy. (Table 3). The highest age class (≥81) showed a statistically significant propensity toward vaccination (OR = 0.05, *p* = 0.003, 95% CI = 0.01–0.36) if compared to the youngest respondents.

## 4. Discussion

The increasing rate of vaccine hesitancy (VH) registered worldwide in the last decade could undermine the efficacy of the ongoing COVID-19 vaccination campaign, with tremendous health and social consequences.

Our study, performed in 1176 subjects affected by type 2 diabetes, demonstrated a not negligible rate (14.2%) of VH toward the COVID-19 vaccine. This finding was quite surprising, as diabetics are considered an “at-risk population, for the clearly established increased prevalence (two- to fourfold) of severe complications and unfavorable outcome of SARS-COV-2 infection with respect to the nondiabetic subjects [6,7,8,11]. In particular, a recent systematic review, meta-analysis, and meta-regression including 45,775 hospitalized COVID-19 patients demonstrated a weighted prevalence of 20% (95% CI: 15–25, I 99.3%) of type 2 diabetes among hospitalized COVID-19 patients, and a weighted prevalence of mortality 82% (1.82 times) higher in diabetes than that in nondiabetic patients (20%, 95% CI: 15.0–26.0; *I*^2^ 96.8% vs. 11%, 95% CI: 5.0–16.0; *I*^2^ 99.3%) [8]. Although the exact etiopathogenic mechanisms remain to be established, several predisposing factors have been postulated, including compromised immune response to viral infections; increased viral binding affinity and entry, and decreased virus clearance secondary to the reduced efficacy of intracellular degradation of bacteria, neutrophil chemotaxis and phagocytosis; and higher cell susceptibility to viral inflammation and damage secondary to protein glycosylation and altered composition of complements [11].

On the other hand, the reluctance of diabetic patients to be vaccinated despite physicians’ recommendations, with consequent unsatisfactory coverage rates, had been previously reported for seasonal influenza [12,13]. Similarly, low influenza vaccination coverage rates were reported in patients with metabolic disorders and with more comorbidities [14]. In agreement with previous studies [12,13], older subjects, men, and those who had been previously vaccinated were more prone toward vaccination, while adverse reactions experienced following previous vaccinations significantly contributed to VH [15,16,17,18].

At the same time, higher education levels were associated with propensity toward vaccination, as demonstrated by previous surveys performed in various populations and countries [16,17,18].

For the tremendous detrimental effects that the diffusion of VH among at-risk populations (considered a priority target) can have on the ability of vaccination campaigns to stem epidemics/pandemics, the assessment of the determinants of VH plays a primary role in the planning of efficacious awareness interventions.

Some limitations for this survey should also be acknowledged. Subjects enrolled in this survey were neither a representative sample of the general Italian population nor of whole population of diabetic patients. In fact, this survey was launched on social network groups/pages, which include only a fraction of all diabetic patients selected for social media utilization skills and level of education/health literacy. In addition, we did not know any of the characteristics of those who declined the invitation to this survey.

In conclusion, our study demonstrated vaccine confidence was higher among those who had been vaccinated against influenza, and the more educated. Safety concerns were the most common declared reason of reluctance toward the COVID-19 vaccine. Studies aimed at assessing the determinants of VH in various population groups are of primary importance to plan sensitization strategies in order to maximize the efficacy of vaccination campaigns.

## Figures and Tables

**Table 1:** Main patient demographic features and attitude toward COVID-19 vaccination (*n* = 1176).

Feature		*n*, %
Age (years)	18–30	142 (12.1)
	31–40	160 (13.6)
	41–50	281 (23.9)
	51–60	283 (24.1)
	61–70	228 (19.4)
	71–80	74 (6.3)
	≥81	8 (0.7)
Gender	Female	860 (73.1)
	Male	316 (26.9)
Education level	Under high school diploma	19 (1.6)
	High school diploma	224 (19.0)
	University degree	583 (49.6)
	Post-university degree	350 (29.8)
Italian region of origin	Central	603 (51.3)
	Northern	366 (31.1)
	Southern	207 (17.6)
Subjects ≥65 years old in the household	Yes	355 (30.2)
	No	821 (69.8)
SARS-CoV-2 infection	Yes	29 (2.5)
	No	1147 (97.5)
Vaccination for influenza (2020–2021)	Yes	812 (69.0)
	No	364 (31.0)
Adverse events to previous vaccinations	Yes	142 (12.1)
	severe	24 (2.0)
	No	953 (81.0)
	No answer	81 (6.9)
Attitude toward COVID-19 vaccination	**Already vaccinated**	**60 (5.1)**
	**Unsure**	**18 (1.5)**
	**Extremely/somewhat likely**	**916 (77.9)**
	**Extremely/somewhat unlikely**	**167 (14.2)**
	*Fear of vaccine side effects*	*126 (10.7)*
	*Previous SARS-CoV-2 infection*	*1 (0.1)*
	*SARS-CoV-2 infection considered not very dangerous*	*1 (0.1)*
	*Against vaccines*	*20 (1.7)*
	*Certified medical contraindications to vaccine*	*19 (1.6)*
	**No answer**	**15 (1.3)**

Bold is for variables of “Attitude toward COVID-19 vaccination”, italics is for subvariables of “Extremely/somewhat unlikely”.

**Table 2:** Predictors of propensity toward the COVID-19 vaccine in diabetic respondents.

Feature		*n*, (%)	*p*
Age (years)	18–30	116 (81.7)	<0.001
	31–40	128 (80.0)	
	41–50	235 (83.6)	
	51–60	232 (82.0)	
	>61	266 (85.8)	
Gender	Women	701 (81.5)	0.022
	Men	276 (87.3)	
Education level	Under high school diploma	10 (52.6)	<0.001
	High school diploma	156 (70.0)	
	University Degree	504 (86.4)	
	Post-university degree	307 (87.7)	
Italian region of origin	Northern	305 (85.0)	0.41
	Central	505 (85.3)	
	Southern	167 (81.5)	
Subjects ≥65 years old in the household	Yes	299 (84.2)	0.29
	No	678 (82.6)	
SARS-CoV-2 infection	Yes	24 (82.8)	0.79
	No	953 (83.1)	
Vaccination for influenza 2020–2021	Yes	746 (91.9)	<0.001
	No	231 (63.5)	
Adverse events to previous vaccinations	Yes	103 (72.5)	<0.001

**Table 3:** Multivariate model of determinants of VH.

Vaccine Hesitancy		OR	*p*-Value	95% CI
	18–30	1		
Age	31–40	0.99	0.985	0.53–1.85
	41–50	0.80	0.448	0.45–1.76
	51–60	0.99	0.980	0.56–1.78
	61–70	1.45	0.263	0.76–2.79
	71–80	0.92	0.858	0.39–2.17
	≥81	0.05	0.003	0.01–0.36
Gender	Female	1.23	0.320	0.82–1.83
Vaccination for influenza 2020–2021		0.16	<0.001	0.11–0.22
Adverse events to previous vaccinations		1.30	0.285	0.80–2.12
Educational level	Under high school diploma	1.00		
	High school	0.21	0.007	0.06–0.66
	University degree	0.15	0.001	0.05–0.47
	Post-university degree	0.71	0.557	0.22–2.27
